# Political games of attack and defence

**DOI:** 10.1098/rstb.2020.0135

**Published:** 2021-04-12

**Authors:** Carsten K. W. De Dreu, Ruthie Pliskin, Michael Rojek-Giffin, Zsombor Méder, Jörg Gross

**Affiliations:** ^1^ Social, Economic and Organizational Psychology, Leiden University, Leiden, The Netherlands; ^2^ Center for Experimental Economics and Political Decision Making, University of Amsterdam, Amsterdam, The Netherlands

**Keywords:** contest theory, decision-making, polarization, ideology, cognitive control, perspective taking

## Abstract

Political conflicts often revolve around changing versus defending a *status quo*. We propose to capture the dynamics between proponents and opponents of political change in terms of an asymmetric game of attack and defence with its equilibrium in mixed strategies. Formal analyses generate predictions about effort expended on revising and protecting the *status quo*, the form and function of false signalling and cheap talk, how power differences impact conflict intensity and the likelihood of *status quo* revision. Laboratory experiments on the neurocognitive and hormonal foundations of attack and defence reveal that out-of-equilibrium investments in attack emerge because of non-selfish preferences, limited capacity to compute costs and benefits and optimistic beliefs about the chances of winning from one's rival. We conclude with implications for the likelihood of political change and inertia, and discuss the role of ideology in political games of attack and defence.

This article is part of the theme issue ‘The political brain: neurocognitive and computational mechanisms’.

## 1. Introduction

Politics often revolve around opposing parties trying to obtain something that others have, such as wealth and influence, or preventing something that others want, such as a change in governmental policy or social practice [1,2]. Although political conflict can take the form of constructive debate and mutual gains negotiation [1–4], it often involves contentious strategies that aim to advance one's own group interests at the expense of another group [2,5,6]. Such contentious behaviour can be personally risky, like taking part in a strike or demonstration, and is collectively costly [3,4,7,8]. Yet, the prospect of winning (or not losing) the conflict may make these costs a worthwhile investment.

How humans trade-off the prospective benefits of (preventing) political change against the immediate cost of conflict remains poorly understood. One prominent line of work has related contentious politics to political ideologies and the associated differences in people's moral conviction and cognitive style [9–21]. Unfortunately, findings are mixed and often limited to specific political issues and contexts [22–25]. In particular, it remains unclear how and why ideological differences translate into decisions that affect both the actor and the political opponent's outcomes. Here, we abstract away from political contexts and ideologies, and instead focus on the generic form of political conflict in which parties either attack or defend a certain *status quo*. We show how using a game-theoretic perspective on political conflict allows us to (i) derive and analyse the fundamental trade-off between winning (or not losing) political competitions on the one hand, and the personal and collective costs on the other; (ii) identify the neurocognitive computations operating during attack and defence; and (iii) better understand the relationship between political ideology and contentious politics.

## 2. Political conflict as a game of strategy

Conflict emerges when the interests and values of interdependent (groups of) individuals are incompatible [3,4]. When political, conflict often revolves around changing the distribution of power and resources between societal groups [3,12,18,19,26,27]. Political conflict thus involves, for example, opposing standpoints on economic policy for taxation and the governance of public goods like education, or on social policy with regard to traditional social and religious values, justice, minority rights or freedom of speech [12,28].

Game theory as developed and used in the economic [4,29], biological [30,31] and political sciences [7,32] models conflict in games of strategy. In its simplest form, a game involves two players or agents, each with two strategies to choose from. Conflict emerges when the strategy combination that one agent prefers is at odds with the strategy combination that the other agent prefers. A prime example is the prisoner's dilemma, in which agents choose whether to ‘cooperate’ or ‘defect’. Both agents prefer the other one to cooperate while defecting themselves. For one agent to achieve their preferred outcome necessarily means that the other agent has to make concessions and *vice versa* [4,33].

Many different games can model conflict, and some games capture certain elements of political conflict better than other games [34]. One such element, which heretofore often remains implicit, is that political conflict is about change advocated by some and opposed by others [18,19,27]. Proponents of political change invest energy in arguing, demanding or organizing political action in the form of demonstrations, strikes and revolutions. Opponents of change, in contrast, ignore or reject demands, shut down demonstrations, organize strikebreakers and imprison (presumed) revolutionaries [5,6]. Examples include the recent debate in the UK between politicians arguing in favour of changing EU-membership (leave) and those defending the *status quo* (remain), the conflicts coinciding with ‘Black Lives Matter’ protests around the world between those calling to abolish monuments to colonialists and those wishing to defend their current status as heroes, and the polarizing conflicts across Europe between reactionary politicians trying to revert back to traditional values and socio-cultural practices, a backward change that more established political parties seek to prevent [35].

**Figure 1.**
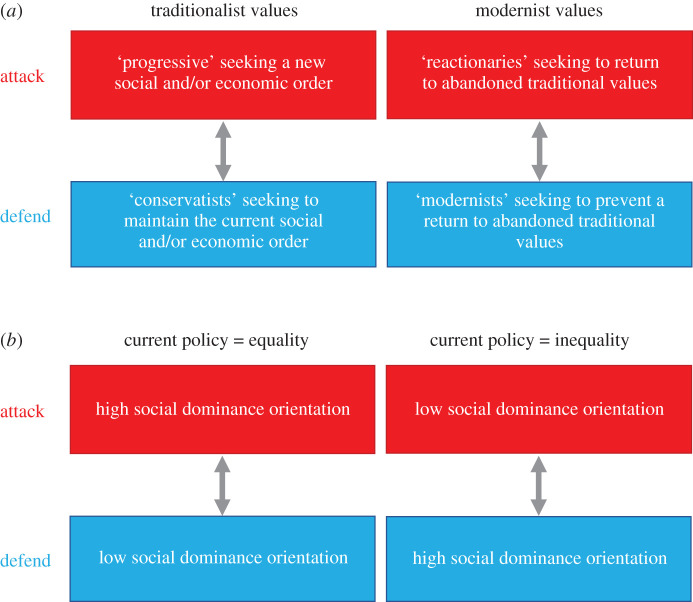
Political conflict over traditionalist versus modernist values and policy for (in)equality. (*a*) Conflict between agents seeking to change versus protect traditionalist and modernist *status quo* (left, progressives versus conservatists; right, reactionaries versus modernists); (*b*) conflict between agents high and low in social dominance orientation seeking to change versus protect current policy for equality and inequality.

In theory, being an advocate or opponent of change is independent of the agents' power and influence, their ‘leftist’ or ‘rightist’ stance, or their positioning on political conservatism or resistance to political change. Liberal ‘progressives’ may advocate new economic principles or social norms that ‘conservatists’ oppose (e.g. greater protections of minority rights, as per the demands of the Black Lives Matter movement), inasmuch as conservatists as ‘reactionaries’ can seek to re-establish abandoned economic principles and social traditions that liberals, as ‘modernists’, try to prevent (e.g. a return to pre-EU ‘glory’ as advocated by supporters of Brexit) (figure 1*a*) [9,13,36–39]. Likewise, agents endorsing inequality between societal groups (i.e. individuals high in social dominance orientation) may fight for changing current socio-economic policy geared at creating equality, a change that is opposed by agents who reject inequality (i.e. individuals low in social dominance orientation) [26,27,40]. Depending on the *status quo*, those high or low in social dominance may seek to revise the governing principles that promote equality or inequality among groups or, alternatively, defend the principles that promote inequality or equality among groups (figure 1*b*).

The element of change that some want and others oppose is modelled in a small family of games, which include the Inspection Game, the Best Shot/Weakest Link Game and the Attacker–Defender Contest (AD-C) ([41–46]; Z Meder, J Gross, CKW De Dreu 2020, unpublished). Each of them models conflict between proponents of change who maximize personal welfare by investing in ‘attack’ and opponents of change who minimize losses by investing in ‘defence’ [42,45,46]. For example, in the AD-C, an attacker (A) and defender (D) each have an endowment *e* from which they can invest *x* in the contest (with 0 ≤ *x* ≤ *e*). Investments model the effort agents expend on political conflict and are non-recoverable, akin to money spent on political campaigns or strikebreakers, and time spent on building alliances and preparing for revolutions and counter-revolutions. When *x*_A_ > *x*_D_, A wins the contest and earns the non-invested resources from D (*e*_D_−*x*_D_). These ‘spoils of war’ are added to A's non-invested resources, yielding a payoff of *r*_A_ = 2*e*−(*x*_A_ + *x*_D_). In this scenario, D earns *r*_D_ = 0, akin to losing voters, territory or political influence to one's political rival. When *x*_A_ ≤ *x*_D_, both players earn their non-invested resources (*e*−*x*_A_, *x*_D_), akin to the *status quo* being preserved at a cost to personal and collective welfare [42,44–46].

## 3. An equilibrium in mixed strategies

**Figure 2.**
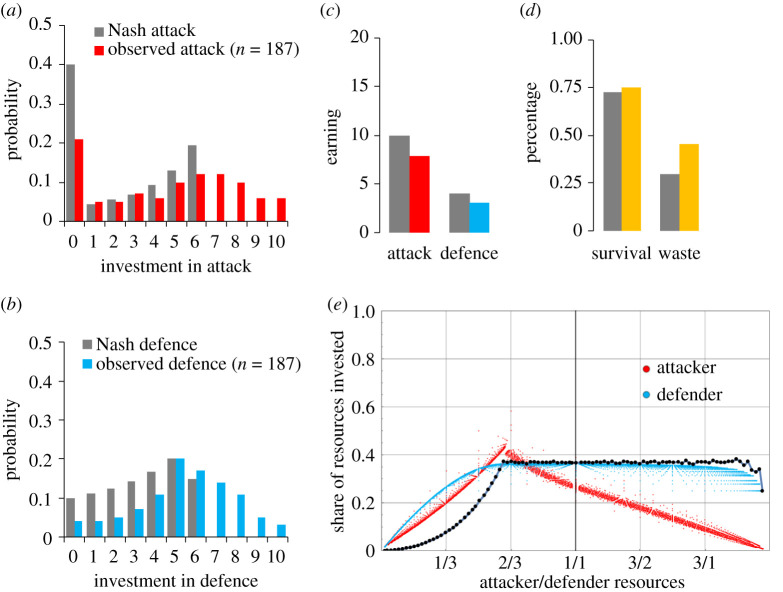
Attacker–defender contest dynamics as predicted (grey bars) and observed (coloured bars). Observations shown here are the weighted aggregate of independent experiments [47,48] in which individuals made a series of investments in attack (defence) each time with a new partner who simultaneously invested in defence (attack). (*a*) Probabilities of investment in attack; (*b*) probabilities of investment in defence; (*c*) welfare consequences; (*d*) likelihood of defender survival and percentage of collective waste; (*e*) expected investments when power differs. Equilibrium analysis shows that conflict is lowest when attackers start out with less than half, and highest when they command about one-third fewer resources than the defender [39]. In the first case, conflict is unlikely to succeed; when resources are equal, or the attackers command a surplus, potential gains are low. Attacker's probability of success (black dots) steeply increases as attacker's gain in relative power up to approximately two-thirds and then remains stationary even when the attacker's power largely exceeds that of the defender.

Players in attacker–defender contests have no clearly advantageous strategy: whether attack or no-attack maximizes the attacker's payoff depends on the strategy chosen by the defender. Likewise, whether defence minimizes the defender's losses depends on the strategy chosen by the attacker [42]. Accordingly, the attacker–defender contest has a single Nash equilibrium in mixed strategies [42,46], and both players should randomize between devoting 0,1,2… resources to the contest, up to a certain highest investment. The share of this highest reasonable investment converges to 1–(1/*e*) ≈ 0.63 [39]. The attacking player's strategy is bimodal, assigning a relatively high probability (close to 1/*e* ≈ 0.37) to refrain from attacking, but making a ‘weak’ attack unlikely. The defender's equilibrium strategy also assigns higher probabilities to stronger defensive actions, but—in contrast with the attacker's strategy—not defending at all is the least likely (an example with *e*_A,D_ = 10 is shown in figure 2; also see [46]).

Because strategic choice has its equilibrium in mixed strategies, both attacker and defender should try to predict their rival's future play and, at the same time, hide their own true intentions from their rival. Players can thus be expected to produce behaviour that is irreducibly uncertain from their rival's point of view and attempt to deceive and mislead their rival by sending wrong signals and engage in cheap talk (*viz*. spreading false information and ‘fake news’) [31,49–51]. At the same time, it is in the defender's best interest to *match* the attacker's strategy (defend when attacked; not defending when not attacked) and in the attacker's best interest to *mismatch* the defender's strategy (attack when defence is low; not attack when defence is high) [42]. Accordingly, defenders can be expected to deter their attackers by (over-)emphasizing their strength and willingness to fight [52,53], while attackers can be expected to signal disinterest and peaceful intent [42]. Since there is no dominant strategy in this game, information plays an important role. Especially, attackers have an interest in revealing credible information about the defender's strength or strategy through, e.g. surveillance or espionage, since their option space is larger and bimodal.

## 4. Power and conflict intensity

The game-theoretic analysis of attacker–defender contests reveals that the outcome of political conflicts depends not only on efforts spent, but also on how convincing each player's signals are and how effective their (counter)intelligence is. In theory, these predictions are independent of the precise magnitude of the endowment [42], but they change when power differences exist in the form of resources available to devote to conflict—when, for example, some agents are wealthier than their rivals, are in the numerical majority, have a more supportive constituency or belong to groups that disproportionally benefit from governing principles for the distribution of resources and influence [47,48,54,55]. By varying the relative resource distribution of the attacker and defender parties, the attacker–defender contest thus can model political conflict between advantaged and disadvantaged groups seeking versus preventing revision of the *status quo*.

Somewhat counterintuitively, less powerful attackers are expected to invest a significant part of their resources into attack. While the likelihood of winning is low, conflict promises large ‘spoils of war’ when facing a strong defender [56,57]. Indeed, in equilibrium, conflict intensity is strongest when attackers have about one-third fewer starting resources than the defender (figure 2*e*). In addition, the more resources attackers have relative to their defender, the lower the share of their resources they should invest. Defenders are expected to be relatively indifferent here and, as a result, attacker success rate remains stationary from the point where attackers are one-third behind their defender, to two-thirds ahead (figure 2*e*). In equilibrium, power differences modulate conflict intensity but barely the extent to which attackers settle the conflict in their favour.

## 5. Neurocognitive mechanisms of attack and defence

Game-theoretic predictions often deviate from what agents actually do [4,8,29,34], and this holds true also for decision-making in attacker–defender contests [42,43,58]. Typically, compared with defence, investments in attack are less frequent and lower overall, yet investments in both attack and defence substantially exceed equilibrium levels: with an endowment of *e* = 10, investments greater than 6 should never occur theoretically yet are frequently observed in laboratory experiments (figure 2*a*,*b*). Because conflict intensity is higher, attackers and defenders earn less than predicted (figure 2*c*), and collective welfare is reduced more than would be expected under rational-choice theory (figure 2*d*) [59–62].

Rational-choice theory is traditionally premised on the threefold assumption that people (i) hold selfish preferences, (ii) have unlimited information processing capacity, and (iii) assume selfishness and unlimited processing capacity in others. Accordingly, one reason why attack and defence in contests are out-of-equilibrium is that people hold *social preferences* [3,8,63]. People with pro-social preferences attach a positive weight to others' welfare, value equality or want to avoid harming others [34,63], whereas people with anti-social preferences attach a negative weight to others’ welfare, value winning and lack empathy for harming others [64–66]. At the neurobiological level, pro-social preferences have been linked to the release of oxytocin, a hypothalamic neuropeptide that functions as both a hormone and neurotransmitter [67–69]. Anti-social preferences, in contrast, have been linked to testosterone, a steroid hormone associated with territorial competition and status-ranking [70,71].

Human decision-making is more self-centred when concerned with minimizing losses rather than maximizing gains [72,73]. Because defence is concerned with preventing losses and must adapt to the aggression levels of attackers [42–46], pro- and anti-social preferences should modulate investment in attack more than in defence. Indeed, pro-social preferences reduce investment in attack but not in defence [61], and when given oxytocin rather than a matching placebo, individuals invest less in attack but not in defence [59]. Whether anti-social preferences and elevated testosterone increase attack more than defence requires testing. Evidence for this hypothesis would fit the finding that prenatal exposure to testosterone associates with more aggressive investments in contest games [71].

A second reason why attack and defence exceed mixed-strategy equilibrium predictions is that people have limited *information processing capacities* and suboptimally compute decision costs and benefits [74,75]. Processing capacity is limited by time constraints and fatigue [76] and related to activity in prefrontal brain regions [77].

**Figure 3.**
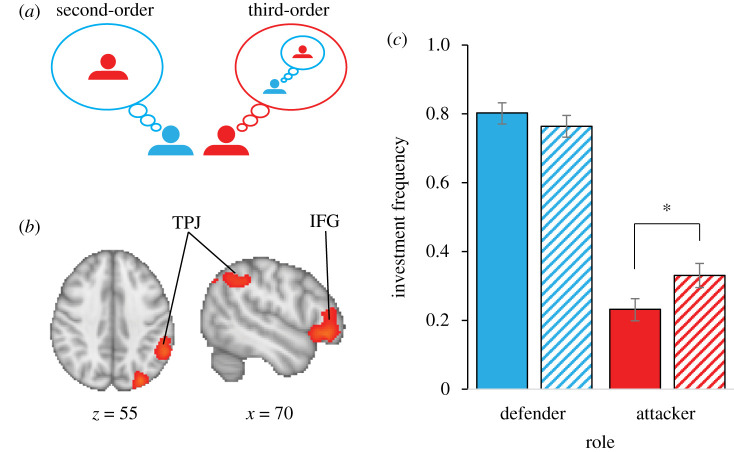
Cognitive hierarchies and neural activity associated with investing in attack and defence. (*a*) In the cognitive hierarchy framework, individuals always assume their rival to be reasoning at one level below themselves. A ‘first-level’ reasoner assumes its rival to be a ‘zero-level’ reasoner (a zero-level reasoner is assumed to make investments randomly, not accounting for the rival's thought process whatsoever), while a ‘second-level’ reasoner assumes its rival to be a ‘first-level’ reasoner, and so on. (*b*) Attacker wins and losses correlate with neural activity in the temporo-parietal junction (TPJ) and inferior frontal gyrus (IFG) (based on [48]). (*c*) Compared with sham-treatment (solid bars), downregulating IFG functionality through theta burst stimulation (hatched bars) increased the frequency of attack but not defence (based on [54]). *p < 0.05. Error bars represent standard errors.

Computational modelling using a cognitive hierarchies framework (figure 3*a*) [78–80] revealed that individuals engage in more sophisticated reasoning about their rival during attack (versus defence) [62,81]. Such sophisticated reasoning during attack (versus defence) was associated with neural activation in the temporo-parietal junction and inferior frontal gyrus, regions typically associated with cognitive control and perspective taking [62] (figure 3*b*). Moreover, temporarily disrupting the functionality of the inferior frontal gyrus with theta burst stimulation increased the frequency of attack (but not defence, figure 3*c*) and reduced attackers' (but not defenders’) tracking of their rival's history of play [60]. It is thus possible that limited information processing capacity leads to less calibrated and more aggressive attack. Indeed, the aggressiveness of attack (but not defence) is associated with shorter decision-latencies [61], and it increases when agents are cognitively taxed [61] and when neural activity in prefrontal structures, commonly associated with value-based decision-making, is reduced [82].

One final reason for out-of-equilibrium attack and defence is that people hold *wrong beliefs* about their rival [83]. Attack benefits from optimism about winning the contest, as ‘[the] hope of victory increases effort, commitment, and persistence in the face of difficulty or threat of failure, and thereby raises the chances of success' [84, p. 49; 85]. In political conflict, overconfidence may be boosted by a party's conviction in its own virtue [86], and by delegitimizing one's rival [87]. By contrast, defenders may benefit from pessimism about their attacker's benign intent and use a ‘better safe than sorry’ strategy (*viz.* hostile attribution bias; [42,88]). Both attacker optimism and defender pessimism may fuel wrong beliefs about their rival's true intentions and escalate investment out of equilibrium.

## 6. Political change and *status quo* inertia

When their attacks are successful and lead to victory, proponents of change gain resources, e.g. in the form of electoral support, influence and wealth, can push their political agenda with greater force, and more likely impose their views on the opposing party. In theory, and all else equal, attackers have a 30% likelihood of being successful. This theoretical prediction is mirrored in findings from both laboratory experiments (figure 2*d*) and archival analyses of revisionist warfare and hostile take-over in industry [42,89].

The low success rates for attackers suggest that political change is unlikely and *status quo* maintenance is the norm. Proponents of change may need frequent attempts to successfully change the *status quo*. When attempts to change the political landscape persistently fail, people may feel helpless [90] and engage in a self-fulfilling process of ‘system-justification’ [91]. This low success rate also suggests that especially proponents of change benefit from an increase in relative power, and may thus invest in building coalitions and alliances and in creating committed and supportive constituencies (also see §8). Given that those who oppose change respond in kind, the attackers' search for power can ignite an arms race for power to coerce and deter [88,92].

In theory, an arms race for power and influence does little to the probability that attackers successfully introduce political change (figure 2*e*). Yet when attackers win, the impact can be quite radical—they shape if not replace defenders’ political interests and values by their own [92–94]. We have shown that victory is more likely when attackers lack pro-social preferences and strategize towards winning rather than maximizing earnings. It follows that political games of attack and defence contribute to a selection of agents, who are low in pro-social preferences, have resources to spend on conflict, are unrealistically optimistic about the likelihood of being victorious, and use their cognitive capacity to compute what it takes to win, rather than to maximize subjective value. At the same time, we showed that games of attack and defence reduce collective welfare (i.e. monetary payoff) by a substantial margin of approximately 40% (figure 2*d*). Compared with collectives that settle their political conflict through mutual gains negotiation and compromise, collectives marked by recurrent games of attack and defence will have reduced relative fitness, risking marginalization and collapse [92–94].

## 7. Political ideology and games of attack and defence

Conceptualizing political conflict as a game of attack and defence sheds light on some difficult issues in the study of political ideology. Extant work has identified psychological differences between people endorsing leftist versus rightist political ideologies. People endorsing more liberal ideology typically favour policy that reduces inequality between societal groups and endorse forward change more than those endorsing more conservative ideology [11,12,18,19,26,27] (also see figure 1*a*,*b*). Our analysis clarifies that people with more liberal political ideologies may, however, not only endorse forward change [11,12], but also, as ‘modernists’, resist backward change [35]. By contrast, people with more conservative ideology not only oppose forward change but may also promote, as ‘reactionaries’, backward change [35]. Regardless of their specific stance on a particular political ideology dimension, people can find themselves in the position of ‘attacker’ or ‘defender’ in ideological conflict (see also figure 1).

Game-theoretically, there is no reason to assume that people with liberal views attack, as progressives, with more effort than those with more conservative views (as reactionaries), or that those with conservative views defend more strongly than people with liberal ideology. In fact, the only solid game-theoretical prediction for political conflicts based on the AD-C is that those seeking to defend the *status quo* will be successful more often than those seeking to alter it, regardless of their ideological position. At the same time, however, there is evidence to suggest that people with more liberal views hold stronger pro-social preferences (*viz.* low social dominance orientation; [95]) than those endorsing more traditional, conservative ideologies [11,14,28,95,96]. Given that pro-social preferences reduce attack more than defence [61], (leftist) progressives should be less willing to engage in contentious politics than (rightist) reactionaries. Progressives may thus be less often victorious than reactionaries, rendering forward change a less likely outcome of political conflict than backward change.

Some studies in political psychology also suggest that a rigid cognitive style and low ‘need for cognition’ are more prevalent among those endorsing rightist rather than leftist ideologies [10,12,17]. We showed that sophisticated reasoning and perspective taking modulate the aggressiveness of attack, but not defence [60–62]. If adhering to conservative ideology is associated with cognitive rigidity more than endorsing liberal ideology, one would expect reactionaries to be relatively more aggressive attackers than progressives, and defence to not differ between modernists and conservatists. Again, all else equal, political conflict is less likely to produce forward change advocated by (leftist) progressives than backward change advocated by (rightist) reactionaries.

Studies relating political ideology to individual physiology and to neural activity in brain regions linked to cognitive control and emotion processing [10,17,23,24] produced variable results [25,26]. Our model provides an explanation in terms of the agent's position in the political conflict that, in theory, can vary independently of political ideology [97] (see figure 1*a*,*b*). Relative to defence, attack associates with more sophisticated reasoning, more activity in brain regions linked to perspective taking and cognitive control [60–62], and appears more under the influence of neurohormones like oxytocin and testosterone [59,70,71]. It follows that the link between political ideology and neural activity in the mentalizing networks or levels of oxytocin and testosterone depends, first and foremost, on the individual's position in a political game of attack and defence. Rather than political views *per se*, it is the structure of the political conflict that drives neurocognitive functioning and whether and how hormones influence cost–benefit analyses, decision-making and the likelihood of winning political power and influence.

## 8. Political games of attack and defence among non-unitary groups

When political conflict revolves around groups rather than individuals seeking versus resisting change, individuals within groups are confronted with a public good provision problem—they share ‘spoils of war’ (and the pride of averting defeat) regardless of whether and how much they personally contributed to attacking the out-group or defending the in-group against outside attack [34,42].

The mixed-strategy equilibrium predictions we developed for games of attack and defence between unitary agents, such as individuals, generalize to intergroup games of attack and defence [42,89]. Experiments confirmed that also individuals nested in groups invest, on average, less in attack than in defence and that the probability of winning the political conflict in intergroup conflict also is approximately 30% [42,82,89,98]. At the same time, we have shown too that within-group cooperation and coordination of collective action become critical to winning, or not losing, the intergroup game of attack and defence [42,89]. In fact, groups seeking change through attack have more difficulty coordinating effective collective action, and face a stronger ‘free-rider’ problem than groups trying to defend the *status quo* [82,89,98]. *Vice versa*, groups defending the *status quo* elicit stronger group-identification and commitment among their members than groups attacking the *status quo* [42,82].

An implication of these findings is that political groups seeking to create change (versus those defending against it) benefit more from institutions such as leadership and communication channels that increase commitment and facilitate the coordination of collective action [42,99]. An interesting possibility awaiting future research is that a shared ideology, alongside strong leader rhetoric, can functionally serve within-group coordination and commitment during attempts to revise the political *status quo* [42,82,98,99], with implications for how splintering within social movements affects the likelihood they will succeed in affecting change.

## 9. Conclusion

The contest game of attack and defence introduced and reviewed here captures some essential features of political conflict. Implementing this model of political conflict in laboratory experiments revealed neurocognitive underpinnings of out-of-equilibrium investment in attack and defence. Deviations from rational selfish play were traced to non-selfish preferences, limited cognitive processing capacities and overly optimistic beliefs. The analytical framework, together with these neurocognitive mechanisms underlying attack and defence, sheds new light on the relationship between political ideology and *status quo* defence. It reveals that the nature of political cognition or sophistication is not necessarily driven only or even chiefly by particular ideological or policy position *per se*, but by one's orientation or stance towards the kind of political arrangements that are currently dominant (i.e. the *status quo*) in a particular political context [97]. As such, the attacker–defender framework offers a mechanistic account for when and why features of the political context, including power differences, the ability to form and break alliances, opportunities for signalling and deception, attempts to manipulate political commitment and ideological beliefs, and cohesiveness among movements, shape political conflict and the likelihood of political change.

## Data accessibility

Illustrations are based on data published elsewhere (with sources being referenced); data and computing scripts underlying these publications can be found there or obtained from the corresponding author.
